# Supplementary material for: The causal relationship between the human gut microbiota and pyogenic arthritis: a Mendelian randomization study
Source: Front Cell Infect Microbiol. 2024 Nov 26;14:1452480. doi: 10.3389/fcimb.2024.1452480 (PMC11629706; doi:10.3389/fcimb.2024.1452480)
Supplement: Supplementary file 1 [file DataSheet1.zip › mendelian test/ebi-a-GCST90027631.csv_leave_one_out.pdf]

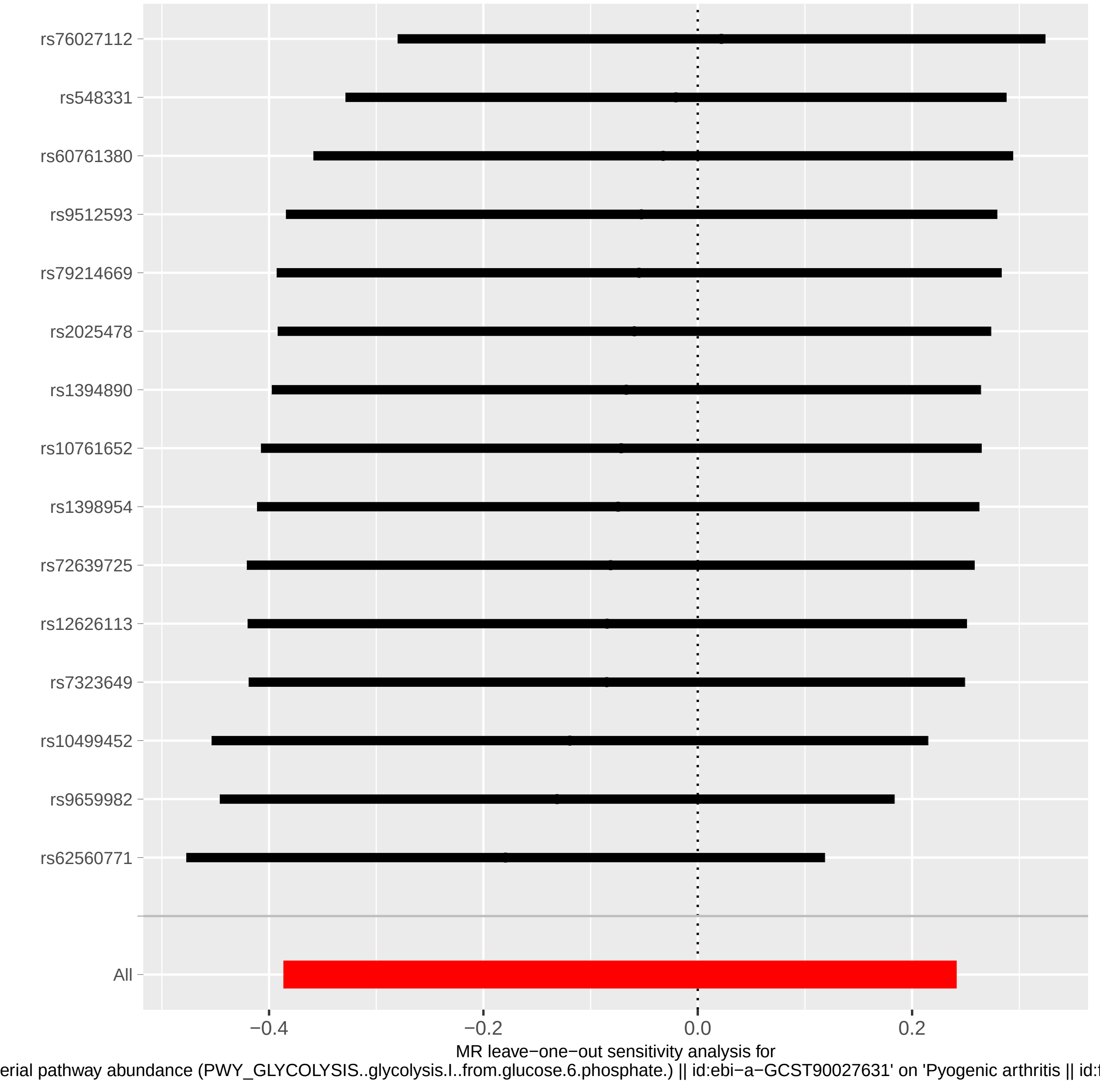
rs76027112
rs548331
rs60761380
rs9512593
rs79214669
rs2025478
rs1394890
rs10761652
rs1398954
rs72639725
rs12626113
rs7323649
rs10499452
rs9659982
rs62560771
All
−0.4
−0.2
0.0
0.2
MR leave−one−out sensitivity analysis for
erial pathway abundance (PWY_GLYCOLYSIS..glycolysis.I..from.glucose.6.phosphate.) || id:ebi−a−GCST90027631' on 'Pyogenic arthritis || id:f
